# Zika Virus Infection Is More Highly Replicative and Transmissible by Extracellular Vesicles in Human than in Mouse Neuronal Cells

**DOI:** 10.3390/ijms262311500

**Published:** 2025-11-27

**Authors:** Kehinde Damilare Fasae, Md Bayzid, Girish Neelakanta, Hameeda Sultana

**Affiliations:** Department of Biomedical and Diagnostic Sciences, College of Veterinary Medicine, University of Tennessee, Knoxville, TN 37996, USA

**Keywords:** ZIKA virus, extracellular vesicles, human SH-SY5Y cells, mouse N2a cells, murine cortical neurons, interferons, TAM receptors

## Abstract

ZIKA virus (ZIKV) infections in human neonates and adults are associated with deleterious effects on brain cognition and neurological disorders. The mechanism(s) of ZIKV infection in neurons and associated neuronal antiviral responses are not fully understood. In this study, we determined the effects of ZIKV infectivity in human neuronal (SH-SY5Y) cells and mouse N2a cells/primary cultures of murine cortical neurons at early and late tested timepoints of infection. The human neuronal cells had higher ZIKV loads compared to the mouse N2a cells, but the viral loads in the murine cortical neurons were between the loads in these two in vitro cell lines. The murine cortical neurons were thought to be more permissive to ZIKV infection, but viral infection kinetics showed a declining trend like that observed in the mouse N2a cells. We noted that infectious extracellular vesicle (EV)-mediated ZIKV infection showed higher viral loads in the SH-SY5Y cells compared to direct infection with laboratory virus stocks. Similar results were obtained with ZIKV infectious EVs in the mouse N2a cells and cortical neurons. In addition, we noted that ZIKV infection significantly induced EV secretion from all three neuronal cells. Also, we found that ZIKV infection modulates the expression of type 1 interferons (IFNs) and entry receptors such as Tyro3, Axl, and MER-TK (TAM). Alongside the increased ZIKV loads in the SH-SY5Y cells, IFN-beta transcript levels and receptors Tyro3/MER-TK were upregulated at early timepoints of infection. Overall, the reduced ZIKV loads and decreasing IFN expression in the mouse neuronal cells suggested a unique murine cellular ability to restrict and limit viral replication. This could be one of the reasons for the unavailability of wild-type mouse models for ZIKV infection. Our data further shows that ZIKV may preferentially infect human rather than murine neuronal cells, and this could be the potential reason for microcephaly in newborns.

## 1. Introduction

Zika virus (ZIKV) is a mosquito-borne flavivirus belonging to the family *Flaviviridae*. In 2015–2016, ZIKV infections emerged in South America and the southwestern parts of the United States, infecting several individuals and predominantly affecting pregnant women [1]. Infections with ZIKV revealed neurological symptoms and complications such as microcephaly in newborns and caused Guillain–Barré syndrome (GBS) in adults [2,3,4]. To cause neurological damage, ZIKV must cross both the maternal placenta and fetal blood–brain barriers to access neurons in developing brains, infect them, and impair neurodevelopmental processes [5,6]. Studies have shown that ZIKV crosses these barriers and targets the neural progenitor cells, including neurons, resulting in neuronal cell death and altering their functions [7]. Additional evidence revealed that ZIKV affects the cytoarchitecture of the brain, disrupting molecular and neurochemical functions and cellular metabolism in neurons [8,9]. However, certain aspects of ZIKV permissiveness within neurons and the infectivity of neural cells are poorly understood. Although ZIKV can infect most of the neural cell types, it differentially infects them according to their differentiation state [2,3,4]. For instance, the differentiation state of human neural precursor cells significantly affects the ZIKV infection outcome, dysregulating the potential Notch signaling pathway and the process of neurogenesis [10]. Similarly, human neuroblastoma cells (SH-SY5Y cells) have increased susceptibility to ZIKV infection when pretreated with a differentiation factor such as retinoic acid [11]. Furthermore, microglia, astrocytes, and oligodendrocytes are perhaps more resistant to ZIKV infection than neurons. An unclear aspect of ZIKV infection in neurons is the unknown mechanism of viral entry and its replication process. ZIKV is believed to enter neuronal cells via entry receptors and adhesion factors, which include TAM receptors, the T-cell immunoglobulin phosphatidyl serine receptor (TIM-4), and DC-SIGN [1,12]. While several in vitro and in vivo models of ZIKV infection have been reported [13,14,15,16,17], no clear data has been shown regarding the preferential infection of neuronal cells by ZIKV. Although some evidence exists for certain neuronal cells, such as progenitor stem cells [11,18], little is known regarding the relative ZIKV infection of human and mouse neuronal cells and cortical neurons. Our study investigated the infectivity, antiviral response, and expression of entry receptors in three neuronal cell types, including human neuroblastoma SH-SY5Y cells, mouse neuroblastoma (N2a) cells, and primary cultures of murine cortical neurons, to delineate the differences and preferences of ZIKV infection. This study also investigated the importance of extracellular vesicle (EV)-mediated ZIKV transmission. The goal of this study is to understand the differences, host specificity, and/or restrictions upon ZIKV infection in humans compared to murine species. This will initiate a delineation in understanding the distinguishing molecular pathways of ZIKV-causing microcephaly in newborns.

## 2. Results

**Figure 1:**
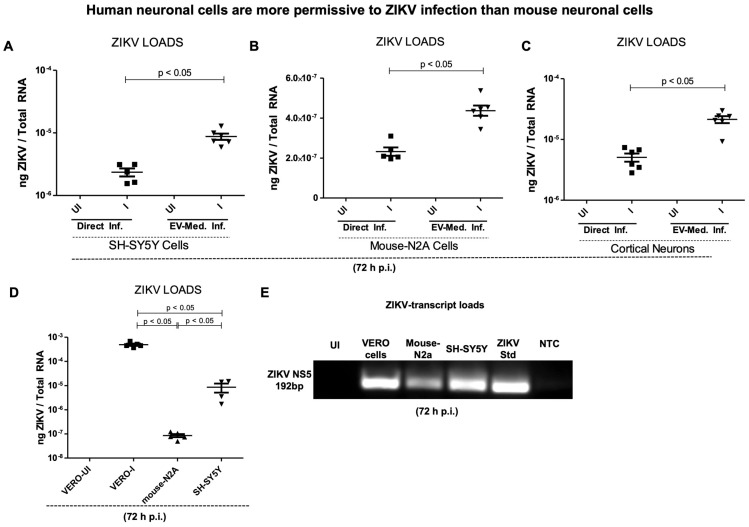
**ZIKV infection in human and mouse neuronal cells.** QRT-PCR analysis showing viral loads (NS5 transcript levels) at 72 h post ZIKV infection in human neuroblastoma SH-SY5Y cells (**A**), in mouse N2a cells (**B**), murine cortical neurons (**C**), and in Vero cells (**D**) infected with direct ZIKV (at 5 MOI) infection for 72 h. In panels (**A**–**C**), all groups were independently infected via EV-mediated infection. Closed squares denote direct infection performed with laboratory ZIKV stocks, and inverted triangles represent EV-mediated ZIKV infection (in Panels (**A**–**C**)). In panel (**D**), all groups of cells were directly infected with ZIKV laboratory stock (5 MOI). Transcript levels for the ZIKV NS5 gene were normalized to total RNA amounts. All treatments had 5–6 independent replicates. *p*-values less than 0.05 are considered statistically significant. (**E**) Agarose gel electrophoresis image showing levels of ZIKV NS5 transcripts in Vero cells, mouse N2a cells, and human SH-SY5Y cells in comparison to the ZIKV NS5 transcript standard used as a positive control for QRT-PCR. The uninfected (UI) group is used as an internal control, and NTC represents no template control for QRT-PCR analysis.

**ZIKV is highly replicative in human neuronal cells compared to mouse neuronal cells and primary cultures of cortical neurons.** An initial evaluation of ZIKV infection was carried out as direct infection (using known titers of laboratory viral stock at 5 MOI) or mediated via infectious EVs in human neuroblastoma (SH-SY5Y) cells, mouse neuroblastoma (N2a) cells, and primary cultures of murine cortical neurons. Since EV-mediated flaviviral transmission has been established from both arthropods to vertebrate hosts [19,20,21,22,23,24] and between neurons of mammalian hosts, we determined the efficiency of the EV-mediated infection in comparison to the infection via laboratory virus stock. In the EV-mediated approach, we isolated EVs from uninfected or ZIKV-infected neuronal cells (infected with 5 MOI of laboratory virus stock for 72 h post infection), and co-incubated them on naïve recipient neuronal cells, respectively. Freshly isolated EVs with unknown titers were used as 20 µL (by volume) from the EV pellet (which was resuspended in 1× PBS solution of 150 µL). Our results show that viral infection was permissive in all the neuronal cells; however, both the SH-SY5Y cells and murine cortical neurons had 1.5 log higher ZIKV loads than the mouse N2a cells (Figure 1A–C). Notably, the EV-mediated transmission of ZIKV consistently appeared to be higher in all the tested neuronal cells when compared to the direct infection via laboratory viral stock.

To understand the EV-mediated infection being higher than the direct infection with ZIKV, we characterized EVs from these three different neuronal cell types according to the ISEV recommendations. Immunoblotting analysis revealed the protein expression of CD9 (EV membrane-enriched marker), HSP70 (heat-shock protein 70; EV lumen/cytosolic marker), and Lactose dehydrogenase (LDH) as NVEP hallmark structure in EVs prepared from the SH-SY5Y human cells, mouse N2a cells, and murine cortical neurons (Supplementary Figure S1A). It was interesting to note that the HSP70 and LDH protein levels were reduced in the ZIKV-infected SH-SY5Y cell-derived EV lysates in comparison to the levels found in EVs derived from the uninfected SH-SY5Y cells (Figure S1A). However, both the mouse N2a cells and cortical neurons showed increased HSP70 protein levels in the ZIKV-infected groups in comparison to the levels noted in EVs derived from the respective uninfected control group of EVs (Figure S1A). The LDH protein levels showed a slight reduction upon ZIKV infection in both the mouse N2a cells and cortical neurons in comparison to their respective uninfected group of EVs (Figure S1A). CD9 expression showed no changes in all three tested neuronal cells in comparison to their respective uninfected group of EVs (Figure S1A). The total protein profile gel image showed the presence of EV markers and NVEPs in these EV lysates (Figure S1A). The nCS1 nanoparticle analysis revealed EV concentrations, numbers, and sizes in these neuronal cells. We quantified EV concentrations (by diluting with 1× PBS/Tween 20 (PBST) in a ratio of 1:2000 or 1:1000) by using Spectradyne’s nCS1^TM^ particle analyzer (from Spectradyne Particle Analysis, Signal Hill, CA, USA) and 65–400 nm diameter cartridges. We found significantly increasing EV concentrations upon ZIKV infection and in all three tested neuronal cells in comparison to their respective uninfected control group of EVs (Figure S1B–D). These instrument-generated graphs reveal the EV numbers and sizes in the neuronal cells. These data indicated that ZIKV infection induces EV secretion, and further revealed that EV markers are variably expressed between these neuronal cells (Figures S1A–D and S2A–F). In addition, we noted that ZIKV loads in African green monkey (VERO) kidney epithelial cells were significantly (*p* < 0.05) higher when compared to the viral loads noted in the SH-SY5Y cells (Figure 1D). We noted that the mouse N2a cells had significantly (*p* < 0.05) lower ZIKV loads in comparison to the viral loads observed in both the Vero cells and human SH-SY5Y neuronal cells (Figure 1D). Our data showed higher ZIKV loads in the infected Vero cells, followed by the SH-SY5Y cells, compared to mouse N2a cells (Figure 1D). Furthermore, DNA agarose gel electrophoresis confirmed the results from QRT-PCR analysis (Figure 1E). We noted reduced viral loads in the mouse N2a cells when compared to the Vero cells and SH-SY5Y cells (Figure 1E). These results showed that ZIKV efficiently infects (in vitro) human neuronal cells compared to mouse neuronal cells.

**Figure 2:**
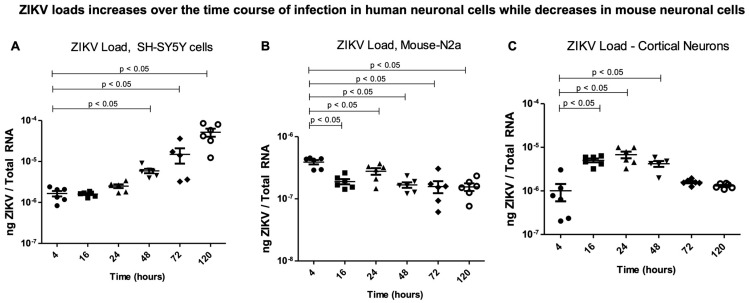
**ZIKV infection kinetics in human and mouse neuronal cells.** QRT-PCR analysis showing viral loads (NS5 transcripts) at tested timepoints of 4, 16, 24, 48, 72, and 120 h post ZIKV infection (with 5 MOI of infection) in SH-SY5Y cells (**A**), mouse N2a cells (**B**), and murine cortical neurons (**C**). Closed circles/squares/triangles/inverted triangles/rhombus/open circles denote direct ZIKV infection via laboratory virus stocks, and at different tested timepoints, respectively. Transcript levels for ZIKV NS5 were normalized to total RNA amounts. All treatments had 5–6 independent replicates. *p*-values less than 0.05 are considered statistically significant.

**ZIKV infection kinetics showed an increasing trend in human neuronal cells.** The differences in the ZIKV loads observed in the human SH-SY5Y cells, mouse N2a cells, and murine cortical neurons prompted us to determine if mouse N2a cells are resistant to ZIKV infection or are incapable of efficient ZIKV replication. We decided to study the infection kinetics of ZIKV in these selected neuronal cells and at different timepoints of infection. We noted that the ZIKV loads increased exponentially over the time course of 4 h to 120 h post infection (p.i.), with a significant (*p* < 0.05) increase observed between 48 h and 120 h in the SH-SY5Y cells (Figure 2A). The mouse N2a cells had significantly (*p* < 0.05) reduced ZIKV loads over the time course of infection compared to the 4 h timepoint p.i. (Figure 2B). The infected cortical neurons initially allowed an increase in ZIKV replication, which persisted until 48 h, but after this timepoint, the ZIKV loads showed a decreasing trend compared to the early tested timepoint of 4 h p.i. (Figure 2C). These data revealed that ZIKV infection kinetics at the tested timepoints post infection are different between the human and mouse neuronal cells.

**Figure 3:**
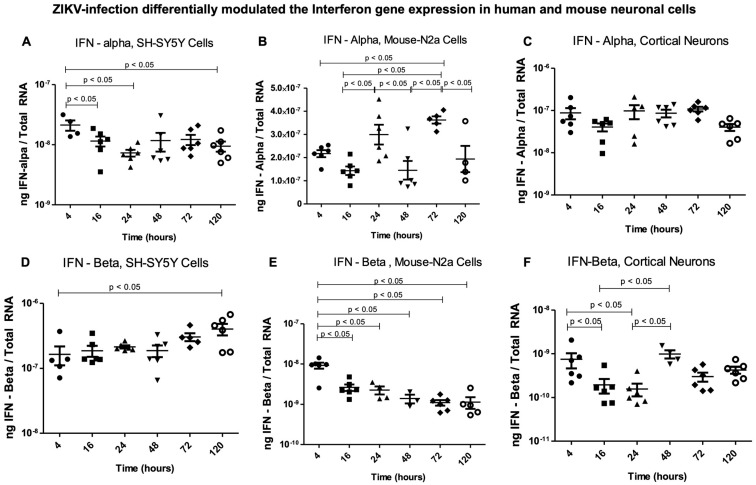
**Antiviral response to ZIKV infection in human and mouse neuronal cells.** QRT-PCR analysis showing IFN-α (**A**–**C**) or IFN–β (**D**–**F**) gene expressions in SH-SY5Y human neuronal cells (**A**,**D**), mouse N2a cells (**B**,**E**), and murine cortical neurons (**C**,**F**) infected with ZIKV (at 5 MOI) at tested timepoints of 4, 16, 24, 48, 72, and 120 h post ZIKV infection. Closed circles/squares/triangles/inverted triangles/rhombus/open circles denote direct ZIKV infection via laboratory virus stocks, and at different tested timepoints, respectively. Transcript levels for ZIKV NS5 were normalized to total RNA amounts. All treatments had 5–6 independent replicates. *p*-values less than 0.05 are considered statistically significant.

**Antiviral gene expression is altered in ZIKV-infected human and mouse neuronal cells.** The induction and activation of the type I interferon system has been known as an important first line of defense (both in vivo and in vitro) against vector-borne flaviviral infections [25]. Therefore, we addressed the increasing (in the SH-SY5Y human neuronal cells) or decreasing (in the mouse N2a cells) ZIKV infectivity by analyzing the associated antiviral IFN response. We determined the expression of IFN-α and IFN-β in these respective human and mouse neuronal cells from 4 h to 120 h post ZIKV infection. IFN-α was significantly (*p* < 0.05) upregulated in the SH-SY5Y cells at the early tested timepoint of 4 h and then was maintained consistently until 120 h (Figure 3A). Expression of IFN-α showed a cycling pattern (of up and down) in its transcript regulation in the mouse N2a cells and over the time course (of 4, 16, 24, 48, 72, and 120 h) post ZIKV infection (Figure 3B). We noted significant (*p* < 0.05) upregulation in IFN-α at 24 h and 72 h post ZIKV infection in the mouse N2a cells (Figure 3B). No significant differences were observed in the gene expression of IFN-α in the murine cortical neurons (Figure 3C). The IFN-β expression showed a significant (*p* < 0.05) increase in the human SH-SY5Y neuronal cells at 120 h (at a later tested timepoint of infection) in comparison to the early 4 h tested timepoint of ZIKV infection (Figure 3D). In comparison to the transcripts noted at 4 h post infection, we observed a significant decrease in IFN-β transcript levels in the mouse N2a cells and at all the other tested timepoints (Figure 3E). In the murine cortical neurons, IFN-β expression was higher at 4 h, followed by a downregulation pattern at the tested timepoints of 16 h and 24 h, and then showed a rapid increase at 48 h and a reduction at 72 h and 120 h post ZIKV infection (Figure 3F). This pattern of cycling expression was like the one observed for IFN-α expression in the mouse N2a cells. Our data showed that upon ZIKV infection, the antiviral response is higher or maintained in the human neuronal cells that had higher ZIKV infection, whereas the mouse neuronal cells revealed an altered pattern of cycling regulation of IFN-α (by the mouse N2a cells) or IFN-β (by the cortical neurons).

**Figure 4:**
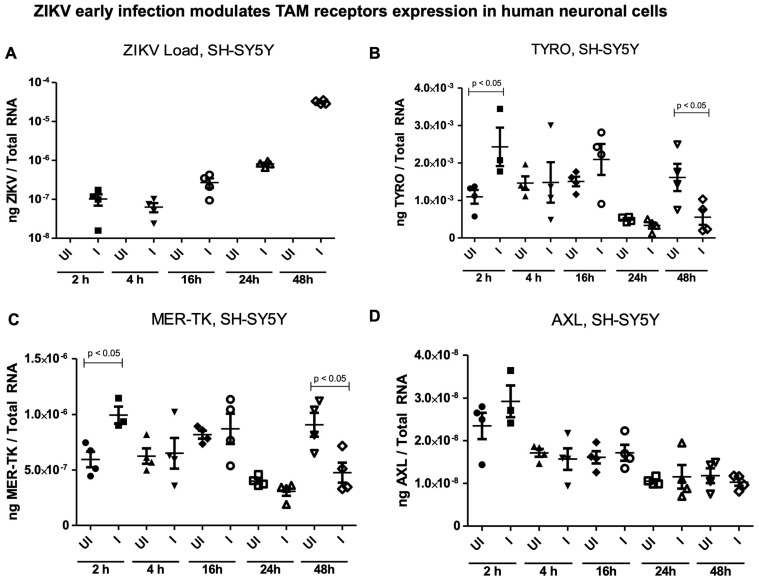
**Effects of ZIKV infection on the expression of TAM receptor at early timepoints in the human SH-SY5Y Cells.** QRT-PCR analysis showing ZIKV NS5 transcripts (**A**) in ZIKV-infected (I) (with 5 MOI at 2, 4, 16, 24, and 48 h post infection) or uninfected (UI) control group, respectively. The gene expressions of TAM receptors, Tyro-3 (**B**), MER-TK (**C**), and AXL (**D**) are shown in both the uninfected and ZIKV-infected groups. Closed circles, triangles, rhombuses, and open squares and inverted triangles denote the uninfected (UI) group. Closed squares, inverted triangles, and open circles/triangles/rhombuses represent the ZIKV-infected (I) group. Transcript levels for ZIKV NS5 were normalized to total RNA amounts. All the treatments had 5–6 independent replicates. *p*-values less than 0.05 are considered statistically significant.

**Figure 5:**
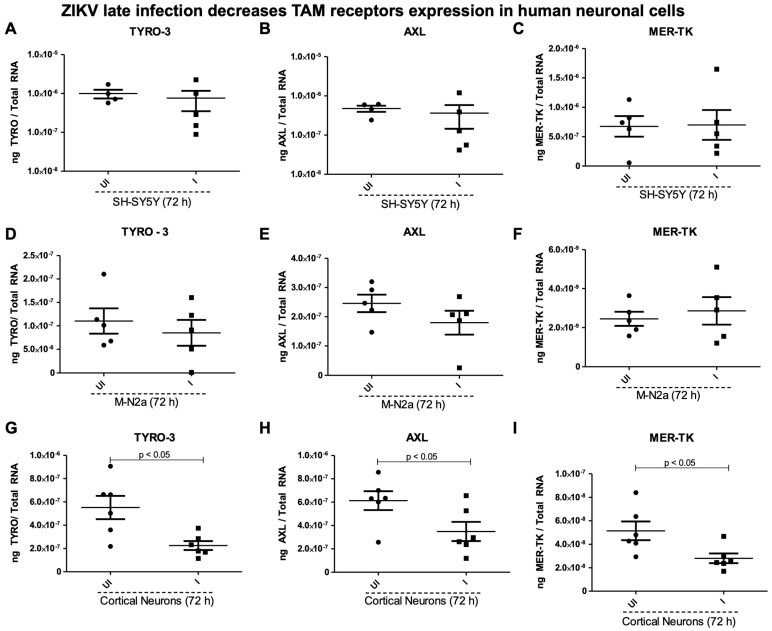
**Effects of ZIKV infection on the expression of TAM receptors at a later timepoint in the human SH-SY5Y cells.** QRT-PCR analysis showing expression of TAM receptors in the SH-SY5Y cells (**A**–**C**), mouse N2a cells (**D**–**F**), and murine cortical neurons (**G**–**I**) at 72 h post ZIKV infection (with 5 MOI). Closed circles denote the uninfected (UI) group and closed squares represent the ZIKV-infected (I) group. Transcript levels for ZIKV NS5 were normalized to total RNA amounts. All treatments had 5–6 independent replicates. *p*-values less than 0.05 are considered statistically significant.

**Effects of ZIKV infection on TAM receptors in human and mouse neuronal cells.** ZIKV’s ability to infect cells, including neuronal cells (used in this study), strongly relies on the presence of specific cellular receptors on the host cell surface [26,27]. TAM receptors (Tyro, AXL, and MER-TK) have been studied upon ZIKV infection in mice and microglial cells [28,29]. We determined the expression of TAM receptors in the SH-SY5Y cells, mouse N2a cells, and murine cortical neurons at the tested timepoints (of 2, 4, 16, 24, and 48 h p.i.) (Figure 4) or at a later tested timepoint of 72 h post ZIKV infection (at 5 MOI) (Figure 5). First, the ZIKV loads were noted to be exponentially increased in the SH-SY5Y neuronal cells at the tested timepoints (of 2, 4, 16, 24, and 48 h p.i.) (Figure 4A). Both Tyro-3 and MER-TK gene expression was significantly (*p* < 0.05) upregulated at 2 h (the early tested timepoint), but at the later tested timepoint of 48 h, we noted significant (*p* < 0.05) downregulation of these two receptors upon ZIKV post infection (Figure 4B,C). No significant differences were noted in AXL gene expression in the SH-SY5Y neuronal cells at any tested timepoints (of 2, 4, 16, 24, and 48 h) post ZIKV infection (Figure 4D). At the later tested timepoint of 72 h, we also noted no significant differences in Tyro-3, AXL, and MER-TK receptor expression in the human SH-SY5Y cells, nor in the mouse N2a cells (Figure 5A–F). However, all the TAM receptors (Tyro-3, AXL, and MER-TK) showed significant (*p* < 0.05) downregulation in the murine cortical neurons at 72 h post ZIKV infection when compared to their respective uninfected control group of cortical neurons (Figure 5G–I). These data showed that TAM receptors are differentially expressed in human and mouse neuronal cells.

## 3. Discussion

ZIKV infection has been associated with severe neuronal cell death and consequent neurological damage in the human brain, including abnormal developmental processes observed in newborns as microcephaly, and Guillain–Barré Syndrome (GBS) in adults [30,31]. Yet, there is no effective treatment available against ZIKV infection of the human brain [32,33]. ZIKV infects several neuronal cell types to varying extents and efficiencies [10,34]. In this study, we investigated the permissiveness and replication of human and mouse neuronal cells to ZIKV infection, examining their antiviral response and receptor expression, to understand how ZIKV colonizes neuronal cells. With Vero cells as an internal control, we report that replication of ZIKV in human neuroblastoma SH-SY5Y cells is highest compared to the viral replication noted in mouse N2a cells and murine cortical neurons at 72 h post infection. Even though ZIKV infection in the SH-SY5Y cells and cortical neurons was comparable, the viral loads in the mouse N2a cells were reduced, thus suggesting a lower permissiveness and replication in the mouse N2a cells. The high susceptibility of SH-SY5Y cells to different ZIKV strains (African MR766 and Brazilian) has been reported [2,35] and is consistent with our current findings. Here, we report that mouse N2a cells are more resistant to ZIKV infection, with lower viral loads when compared to human SH-SY5Y neuronal cells.

In addition, our study showed increased ZIKV loads in all these neuronal cells when EV-mediated infection was performed compared to the infection brought with laboratory viral stocks (as MOI 5), thus indicating successful transmission of ZIKV from EVs to naïve cells. Published studies have shown that EV-mediated infection with unknown titers of infectious EVs isolated from tick cells, mosquito cells, and neuronal cells brings in enhanced viral loads (of Langat virus (LGTV), dengue, and ZIKV) in naïve recipient tick/mosquito/neuronal cells, and alteration of these EVs (by treatment with temperatures, pH and salts) affects their ability to transmit those infectious loads contained in EVs [19,21,23,24]. We believe that infectious EVs bring in less than 50% of infection when compared to the infection with 5 MOI of known titers of laboratory viral stocks (as determined by viral plaque assay) [19,21,23,24]. A low number of infectious particles is perhaps associated with EVs isolated from infected cells. Our previous data showed that full-length flaviviral RNA genomes contained in an infectious EV are sufficient to establish the highest infection in naïve recipient cells [24,36,37]. The increased ZIKV loads noted in EV-mediated infection are higher due to enhanced levels of full-length viral RNA genomes, polyproteins, and perhaps viral particles packaged into infectious EVs. Also, our data show significantly higher EV concentrations upon ZIKV infection in all three neuronal cells, further supporting enhanced EV-mediated transmission. The EV-enriched membrane marker CD9 showed no differences, further suggesting that ZIKV might not commonly prefer CD9 in this preferential infection and transmission in neuronal cells. However, the decrease in protein levels of HSP70 chaperone and LDH in SH-SY5Y cells is in question, and we believe that future research on this finding will be important.

An examination of ZIKV infection in the human and mouse neuronal cells at earlier tested timepoints of post infection revealed the trends of viral replication or dissemination within these neuronal cells. The ZIKV loads increased in the SH-SY5Y cells over the time course of infection. At the 48 h timepoint post infection, we noted reduced ZIKV loads in mouse N2a cells, while an initial increase was observed in murine cortical neurons from 16 h to 48 h. Our results indicated enhanced viral loads in both the SH-SY5Y cells and cortical neurons at early timepoints (2 h to 48 h). However, similar levels of viral loads were not observed in the mouse N2a cells. We noted that at the initial timepoint of 4 h post ZIKV infection, all the neuronal cell lines showed similar viral loads (with a scale of 10^−6^); however, over the time course of infection, the mouse N2a cells decreased the ZIKV loads, perhaps by lowering viral replication. The murine cortical neurons showed an increase at 16 h and 24 h; however, reduced ZIKV loads were noted over the later tested timepoints of 48 h, 72 h, and 120 h post infection. In the SH-SY5Y cells, ZIKV loads steadily increased over the time course of infection and suggested efficient replication in those human cells when compared to the mouse neuronal cells. These data suggested that the mouse N2a cells were perhaps resistant to ZIKV infection or were incapable of efficient ZIKV replication. Also, lower EV production and release in the mouse neuronal cells in comparison to the human cells further suggested that higher transmission rates from infected cells perhaps enhance the viral replication in naïve recipient human cells. To understand why the ZIKV loads in the mouse N2a cells were significantly reduced at every tested timepoints when compared to the human SH-SY5Y cells, we determined the type 1 interferon responses (transcript levels of both IFN-α and IFN-β) in the human and mouse neuronal cells. The maintained IFN-α expression in the human SH-SY5Y cells and cortical neurons suggests a requirement for this interferon response throughout ZIKV infection. The up- and downregulation or cycling of IFN-α expression in the mouse N2a cells suggested a spatio-temporal regulation in the antiviral response. IFN-β transcripts showed an increasing trend in the SH-SY5Y cells at later tested timepoints and indicated a maintenance of antiviral response upon ZIKV infection. In contrast, the time-dependent downregulation of IFN-β transcripts in the mouse N2a cells suggests that the IFN-β response is not activated/needed in these cells or may be inhibited at the early timepoint post ZIKV infection. The up- and downregulation or cycling of IFN-β expression in the murine cortical neurons suggested a spatio-temporal regulation of this antiviral response upon ZIKV infection again. Indeed, almost all non-structural (NS) proteins of ZIKV, especially the NS5 protein, have been shown to interfere with type I-IFN production via the inhibition of the IFN-β promoter [38,39,40]. However, this leads to the question of the significant reduction in the ZIKV loads in the mouse N2a cells. One reason could be due to a robust immune system, as observed in wild-type mice infection with ZIKV [41]. In mouse studies (in vivo), the immune system activation and production of IFNs restrict the viral propagation and dissemination. Yet, we have previously observed that mouse N2a cells are permissive to other viral infections, such as the Langat virus (LGTV), producing substantially infective titers and higher viral loads [23].

Furthermore, we determined the expression of receptors mediating ZIKV entry to rule out the possibility of defective entry and to account for the substantially lower viral loads noted in the mouse neuronal cells. By considering the TAM (Tyro3, Axl, and MER-TK) protein tyrosine kinase family of receptors, which are reported as candidates for ZIKV entry [21,22,23], we observed significant differences in TAM receptors (Tyro3 and MER-TK) expression in the SH-SY5Y cells at the early tested timepoint of 2 h post ZIKV infection. Also, Axl showed an increase in expression upon ZIKV infection. Variable expression of these two receptors was noted at the other tested timepoints of 4 h, 16 h, and 24 h post infection in the SH-SY5Y cells. However, these two receptors were significantly downregulated in the SH-SY5Y cells at the later timepoint of 48 h post ZIKV infection. The downregulation of these two receptors indicated the exocytosis of ZIKV, perhaps via the EV-mediated exit process. Overall, an assessment of the early timepoint of ZIKV infection in the SH-SY5Y cells revealed upregulation of Tyro3 and MER-TK at 2 h and downregulation at 48 h post infection. We noted that ZIKV does increase the expression of AXL at 2 h, but we noted a reduction over the time course of post infection. No significant differences were noted at 72 h post ZIKV infection, thus suggesting that TAM receptors could play a potential role in the entry of ZIKV and may not influence the exit process. These data also suggest that ZIKV utilizes the entry receptors Tyro3 and MER-TK in the first 2 h of infection. The efficient increase in viral replication is perhaps the key to enhanced ZIKV loads in SH-SY5Y human cells. In conclusion, our results show that ZIKV replicates efficiently in human SH-SY5Y cells compared to mouse N2a cells and cortical neurons, and our current findings are consistent with findings from other studies [2,10,34]. Efficient ZIKV entry and infection of SH-SY5Y cells could be the outcome of deleterious effects of ZIKV infection in the human brain. The inefficiency of viral replication in mouse N2a cells is perhaps associated with type 1 interferon responses or TAM receptors. We also believe that there could be other factors within the mouse neuronal cells that might prevent, restrict, or limit viral infections, and may be important in the fight against ZIKV-mediated neuronal damage. Neuronal primary cultures of cortical neurons (as identified subset of neurons) are targeted by ZIKV infection, and this partially explains the incidence of GBS in human patients. Our study suggests that ZIKV preferentially infects human neuronal cells over murine neurons, and this could be one of the reasons for the development of microcephaly in newborns.

## 4. Materials and Methods

**Neuronal cell lines, primary cultures of cortical neurons, and ZIKV infection.** Human neuroblastoma SH-SY5Y cells (Cat Number: CRL-2266) and mouse neuroblastoma (N2a) cells (Cat Number: CCL-131) were obtained from American Type Culture Collection (ATCC, Manassas, VA, USA), and primary cultures of murine cortical neurons were used in this study. Mice studies were approved by the Institutional Animal Care and Use Committee (IACUC) and adhere to the recommendations of the NIH ‘Guide for Care and Use of Laboratory Animals (USA)’. Husbandry and euthanasia were performed based on the institutionally approved animal protocol (#2890-0522, Approval Period: 9 May 2025 until 8 May 2028). Cortical neurons were isolated from embryonic brains of day 16 (E16) gestational female mice (obtained from Jackson Laboratories, Bar Harbor, ME, USA). Plates seeded with cortical neurons were pre-coated with 5 µg/mL of Poly-L-Lysine in 1× PBS (obtained from Thermo/Fisher Scientific, Waltham, MA, USA). Cortical neurons were grown in complete neurobasal medium containing 10% heat-inactivated fetal bovine serum (FBS) (Obtained from VWR, Radnor, PA, USA) and cultured according to the methods described in our published studies [23,24,42]. All primary cultures were tissue-derived from embryonic brains collected from six-week-old gestational female mice. Neuroblastoma cell lines (human SH-SY5Y or mouse N2a cells) and Vero cells (monkey kidney epithelial cells, Cat Number: CCL-81, obtained from ATCC, Manassas, VA, USA) were cultured in Dulbecco’s Modified Eagle’s Medium (DMEM, containing 10% FBS and 1% Penicillin-Streptomycin, obtained from Thermo/Fisher Scientific, Waltham, MA, USA) and propagated at 37 °C with 5% CO_2_ and as described [19,23]. ZIKV strain PRVABC59 (Human/2025/Puerto Rico; Cat Number: NR-50240; obtained from BEI resources, Manassas, VA, USA) was used in this study. Viral stock titers were determined in C6/36 mosquito cells (Cat Number: CRL 1660; obtained from ATCC, Manassas, VA, USA). Neuronal cells were harvested and seeded at a density of 2 × 10^5^ cells per well of 12-well plates. Cells were infected with ZIKV (at a multiplicity of infection (MOI) of 5) at different timepoints ranging from 2 h to 72 h post infection. Cell lysates were collected post infection at the indicated timepoints and considered for further analysis.

**Extracellular vesicle (EV) isolation and quantification.** EVs were isolated as per the published protocols [23,24]. EVs/exosomes were isolated from uninfected/ZIKV-infected neuronal cell culture supernatants and by the differential ultracentrifugation method [23,24]. Briefly, the human SH-SY5Y cells, mouse N2a cells, and murine cortical neuronal cells were seeded at densities of 5 × 10^5^ in their respective complete media (DMEM medium for the SH-SY5Y cells and mouse N2a cells, or neurobasal media for primary cultures of the cortical neurons) with 10% FBS for EV isolation. After overnight incubation of these neuronal cells in their respective media (please note that cortical neurons are non-dividing cells, unlike SH-SY5Y cells and mouse N2a cells), all the neuronal cells were infected with ZIKV (MOI 5, for each cell line) for 72 h p.i. For murine cortical neurons, on the next day, half of the media was replaced with no FBS (to eliminate glial cells’ growth in neuron cultures), followed by ZIKV infection. Cell culture supernatants from the uninfected or ZIKV-infected respective groups were spun at 100,000× *g* for 70 min. EV pellets were washed with ice-cold 1× PBS (with another spin at 100,000× *g*, for 70 min). Freshly prepared EV pellets from the respective uninfected or ZIKV-infected neuronal cells were resuspended in 1× PBS and co-incubated with the same group of naïve recipient cells for 72 h. Neuronal EV purity, size, characterization, and enriched markers have been described in our previous studies [22,43,44]. As per the ISEV recommendations, we performed immunoblotting analysis to show the proteins of categories 1, 2, and 3 as EV hallmarks and to assess the presence of NVEPs in these EV preparations. Pelleted EVs were collected in cold 1× PBS and as multiple replicates for each group. Isolated EVs (resuspended in 1× PBS) were briefly stored or quantified immediately with the nCS1 nanoparticle analyzer (from Spectradyne Particle Analysis, Signal Hill, CA, USA) and by following the manufacturer’s recommendations or as described [22,43,44]. Isolated EVs from the neuronal cell culture supernatants were diluted (1:2000 or 1:1000) in 1% Tween-20 prepared in filtered 1× PBS. Diluted samples (5 µL) were loaded onto the nCS1 microfluidic cartridge TS-400 with a size range of 65–400 nm (in diameters) (obtained from Spectradyne Particle Analysis, Signal Hill, CA, USA). The loaded cartridges were inserted into the nCS1 analyzer instrument to quantify EVs from each sample. Data was collected from four independent replicates and processed as described [22,43,44].

**RNA extractions, cDNA synthesis, and QRT-PCR analysis.** Following the manufacturer’s instructions, total RNA was extracted from neuronal cells by using Aurum total RNA mini kit (Bio-Rad, Hercules, CA, USA). RNA concentrations were determined using BioTek Cytation 7 cell imaging Multimode Reader System (Bio-Tek/Agilent Technologies, Santa Clara, CA, USA). The cDNA was synthesized using the iScript cDNA synthesis kit (Bio-Rad, Hercules, CA, USA), and QRT-PCR (Quantitative real-time PCR) was performed using iQ-SYBR Green master mix (obtained from Bio-Rad, Hercules, CA, USA/QuantaBio, Beverly, MA, USA/VWR, Radnor, PA, USA) or Maxima SYBR/R (Thermo/Fisher Scientific, Waltham, MA, USA), and CFX Opus instrument (Bio-Rad, Hercules, CA, USA). Primers were used from either previously published study [24] (e.g., to determine ZIKV loads) or oligonucleotides were newly designed (and obtained from IDT Custom Oligos, Research Triangle Park, NC, USA), prepared as dilutions, and used in this current study. Primer sequences used in this study are shown in Table 1. Data obtained from QRT-PCR were analyzed using the standard curve method, in which 10-fold serial dilutions were used to establish standards ranging from 1 to 1 × 10^5^ ng, where actual gene quantities were determined relative to different known standard concentrations. Accordingly, viral loads or gene transcripts were normalized to total RNA concentrations. The samples were run in duplicate and as 5–6 replicates. In all the QRT-PCR runs, NTC (no template controls) are included as negative controls.

**Immunoblotting Analysis.** Immunoblotting was performed as previously described [23,24]. Briefly, 1–2 × 10^6^ respective neuronal cells were seeded in a T-25 flask for overnight incubation followed by ZIKV infection (at MOI 5 for 72 h p.i.). EVs were isolated by the differential ultracentrifugation method. Total protein lysates were collected from the EVs and resuspended in modified RIPA lysis buffer, followed by total protein amounts estimation by Bradford assay (BCA kit from Pierce™ Bradford Protein Assay Kit, Thermo Fisher Scientific, Waltham, MA, USA). Total proteins from the respective uninfected or ZIKV-infected EV lysates were then processed for immunoblotting analysis. Total lysates prepared from the EVs (15 μg from UI or ZIKV-infected groups of SH-SY5Y or mouse N2a cells or 8 μg from cortical neurons) were separated on to 12% SDS-PAGE gels (under reducing conditions). Total protein profile gel image served as control. After gel electrophoresis, total proteins were transferred on to nitrocellulose membranes and blocked for overnight in 5% milk and 1× TBST buffer containing Tween-20 detergent (at 4 °C). Membranes were probed with rabbit monoclonal antibodies against CD9 (Cell Signaling Technologies, Danvers, MA, USA), HSP70 or LDH (obtained from ABclonal Science Inc., Woburn, MA, USA) and at dilutions of 1:1000 for overnight at 4 °C. After washes, blots were incubated with HRP-conjugated rabbit secondary antibody (at 1:5000 dilutions, at RT for 1 h) (obtained from ABclonal Science Inc., Woburn, MA, USA). The blots were developed by incubating with ECL reagents (Clarity™ Western ECL Substrate Kit, VWR, Radnor, PA, USA) and imaged/documented with Chemidoc MP imaging system with ImageLab Touch software Version 2.4.0.03 (Bio-Rad, Hercules, CA, USA) and according to the manufacturer’s recommendations.

**Statistical Analyses.** Data sets were analyzed using the GraphPad Prism 6 software (from Dotmatics, Boston, MA, USA) and Microsoft Excel. Unpaired *t*-test or one-way ANOVA with Tukey’s or Dunnett’s post hoc multiple comparison tests were used for all analyses. The mean ± SD was used to plot all graphs, and statistical significance was determined as *p* < 0.05. All data points presented in graphs represent the biological replicates from three independent experiments, respectively.

## Figures and Tables

**Table 1:** Oligonucleotides used in this study.

Gene Name	Forward Primer (5′-3′)	Reverse Primer (5′-3′)
Mouse IFN-Alpha	GGACTTTGGATTCCCGCAGGAGAAG	GCTGCATCAGACAGCCTTGCAGGTC
Mouse IFN-Beta	AACCTCACCTACAGGGCGGACTTCA	TCCCACGTCAATCTTTCCTCTTGCTTT
Human IFN-Alpha	GGTAGCAGGAGGACCTTGATG	GGAGGACAGGGATCCTTTCAG
Human IFN-Beta	CTCAAGGACAGGATGAACTTTG	TTTTCTTCCAGGACTGTCTTCA
Mouse AXL	TGGAAGGTCAGCTCAATCAG	CTGTCAGAGCCCTGAAAACA
Mouse Tyro 3	AAGTGGCAGTGAAGATGCTG	GAATGGGGAGACGACCTTTA
Mouse MER-TK	CCCACAATGACAAAGGACTG	ATTACTCAGCCGGTCAGCTT
Human AXL	AGGTCAGAGCTGGAGGATTT	GGGAATAGAGGAGGAAGCTG
Human Tyro 3	GTCCTGGGTTCAAGACAATG	GTTAGCACACCAACCACCAC
Human MER-TK	AAGTCAGCATCCGTAACAGC	CACTGCAGACCAGCCTATTT

## Data Availability

The original contributions presented in this study are included in the article/Supplementary Material. Further inquiries can be directed to the corresponding author.

## Supplementary Materials

The following supporting information can be downloaded at https://www.mdpi.com/article/10.3390/ijms262311500/s1.
