# Supplementary material for: Zika Virus Infection Is More Highly Replicative and Transmissible by Extracellular Vesicles in Human than in Mouse Neuronal Cells
Source: Int J Mol Sci. 2025 Nov 27;26(23):11500. doi: 10.3390/ijms262311500 (PMC12692108; doi:10.3390/ijms262311500)
Supplement: Supplementary file 1 [file ijms-26-11500-s001.zip › ijms-3842198-supplementary.pdf]

Zika virus infection is highly replicative and transmissible by Extracellular Vesicles in human than in mouse neuronal cells

Kehinde Damilare Fasae [1], Md. Bayzid, Girish Neelakanta [1], and Hameeda Sultana [1,*]

[1] Department of Biomedical and Diagnostic Sciences, College of Veterinary Medicine, University of Tennessee, Knoxville, TN, USA.

Supplemental Figures

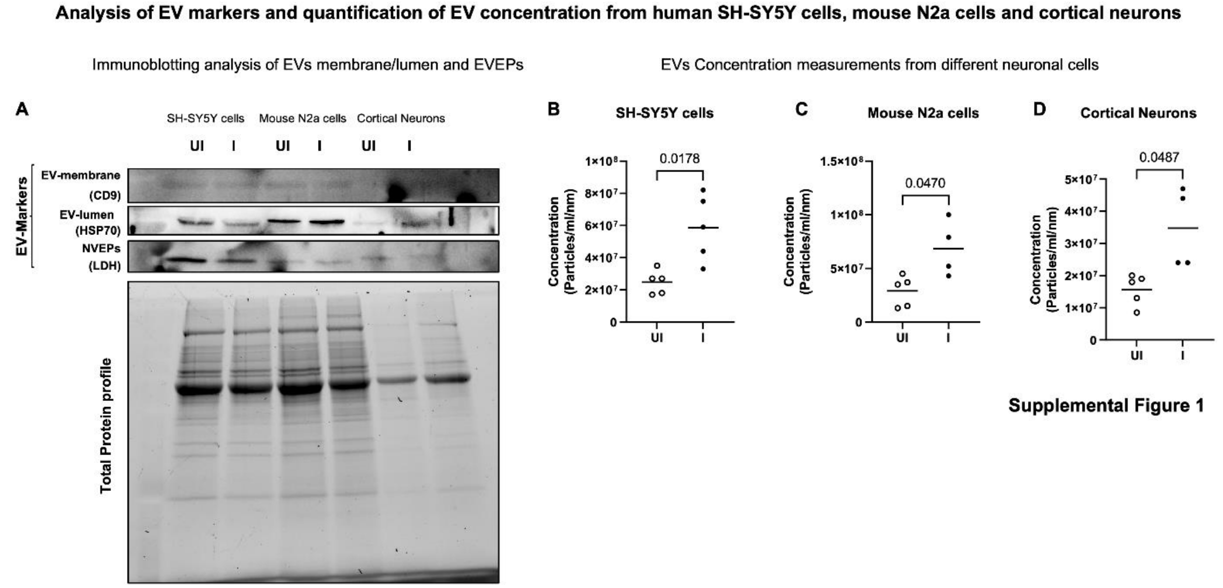

Supplementary Figure S1. Analysis of EV markers and quantification of EV concentrations from human and mouse neuronal cells. (**A**) Immunoblotting analysis showing protein levels of EV membrane enriched marker CD9, EV lumen enriched marker HSP70 and NVEP structure such as LDH in neuronal EVs derived from human SH-SY5Y cells, mouse N2a cells and cortical neurons. Total protein profile gel image from EV protein lysates from neuronal cells served as control. (**B**) Measurement of EV concentrations from uninfected and ZIKV-infected SH-SY5Y cells, or mouse N2a cells

(**C**) or murine cortical neurons (**D**) is shown. Each circle represent an independent sample, and open circles indicate uninfected and closed circles denote ZIKV-infected groups. P values less than 0.05 are considered statistically significant.

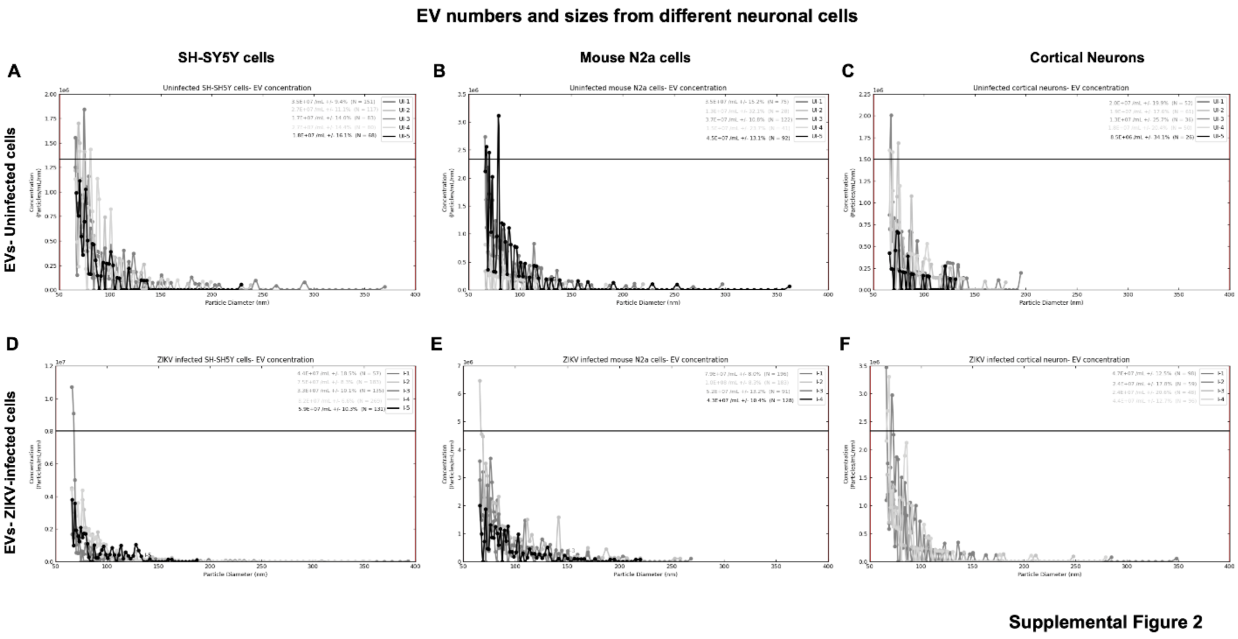

Supplementary Figure S2. Quantification of EV numbers and sizes from human and mouse neuronal cells. Graphical representations (generated by the instrument) showing neuronal EVs quantification by nCS1 analyzer in uninfected (**A**–**C**) and ZIKV-infected SH-SY5Y cells (**A**,**D**), mouse N2a cells (**B**,**E**) and murine cortical neurons (**C**,**F**). Independent replicates (in duplicates) were considered for measuring EVs

concentration. Diameter of each particle and number of EVs was analyzed by the instrument and at the same time.
